# An aryl hydrocarbon receptor induces VEGF expression through ATF4 under glucose deprivation in HepG2

**DOI:** 10.1186/1471-2199-14-27

**Published:** 2013-12-12

**Authors:** Jun Terashima, Chie Tachikawa, Kenzo Kudo, Wataru Habano, Shogo Ozawa

**Affiliations:** 1 Department of Pharmacodynamics and Molecular Genetics, School of Pharmacy, Iwate Medical University, 2-1-1 Nishitokuda, Yahaba-CHO, Siwa-Gun 028-3694, Iwate, Japan; 2 Department of Pharmacy, Iwate medical University Hospital, 19-1 Uchimaru, Morioka 020-8505, Iwate, Japan

**Keywords:** Aryl hydrocarbon receptor (AhR), Vascular endothelial growth factor (VEGF), Angiogenesis, Activating transcription factor 4 (ATF4), Human hepatocellular liver carcinoma cell

## Abstract

**Background:**

Aryl hydrocarbon receptor (AhR) not only regulates drug-metabolizing enzyme expression but also regulates cancer malignancy. The steps to the development of malignancy include angiogenesis that is induced by tumor microenvironments, hypoxia, and nutrient deprivation. Vascular endothelial growth factor (VEGF) plays a central role in the angiogenesis of cancer cells, and it is induced by activating transcription factor 4 (ATF4).

**Results:**

Recently, we identified that glucose deprivation induces AhR translocation into the nucleus and increases *CYP1A1* and *1A2* expression in HepG2 cells. Here, we report that the AhR pathway induces VEGF expression in human hepatoblastoma HepG2 cells under glucose deprivation, which involves ATF4. *ATF4* knockdown suppressed VEGF expression under glucose deprivation. Moreover, *AhR* knockdown suppressed VEGF and ATF4 expression under glucose deprivation at genetic and protein levels.

**Conclusions:**

The AhR-VEGF pathway through ATF4 is a novel pathway in glucose-deprived liver cancer cells that is related to the microenvironment within a cancer tissue affecting liver cancer malignancy.

## Background

The aryl hydrocarbon receptor (AhR) is a well-known transcription factor that is involved in the detoxification response to pollutants and intrinsic biological processes of multicellular organisms. AhR forms a complex with AhR nuclear translocator (ARNT) and regulates the expression of drug-metabolizing enzymes.

AhR has been shown to be activated by xenobiotics including benzo[a]pyrene and 2,3,7,8-tetrachlorodibenzo-*p*-dioxin, and it induces the expression of genes containing an XRE domain in their promoter region [1,2]. The genes, which are activated by AhR, include loci encoding the cytochrome P450 family, which is responsible for the phase I detoxification response, enzymes metabolizing endobiotics and xenobiotics, and other molecules that function in cell differentiation [3-7]. In addition, AhR is one of the stress response molecules in mouse hepa1c1c7 cells; crosstalk between AhR and nuclear factor erythroid-derived 2-related factor 2 (Nrf2) mediates the oxidative stress response through NAD(P)H dehydrogenase quinone 1 (NQO1) [8]. Miao et al. demonstrated that Nrf2 transcription is directly modulated by an activated AhR [9]. AhR has been studied not only as a mediator of chemical toxicity but also as a regulator of vascular development or angiogenesis [10]. Vascular endothelial growth factor (VEGF) is a potent angiogenic factor that plays a central role in angiogenesis, and it is a recognized gene marker for angiogenesis [11]. VEGF expression is induced by various environmental stresses, nutrient deprivation [12], and hypoxia [13]. Glucose deprivation, one of the nutrient deprivations of MCF-7/ADR cells, induces the expression of cellular homologs of oncogenes and angiogenic factors [14,15]. These results support the hypothesis that glycolytic metabolism is associated with cancer malignancy [16]. In human hepatoblastoma HepG2 cells, glucose deprivation induces VEGF expression [17]. We reported that the nuclear localization of AhR was induced in HepG2 cells in low glucose conditions [18]. The AhR localization induced not only *cytochrome P450 family 1* member *A1* (*CYP1A1*) and *1A2* expression but also *Nrf2* expression. In addition, AhR mediates TCDD induced VEGF expression [19] and relates to angiogenesis in mouse [20]. In this study, we report a novel pathway that induces VEGF expression in HepG2 cells in response to glucose deprivation. The response to glucose deprivation that is mediated by AhR induces VEGF expression through activating transcription factor 4 (ATF4) expression in HepG2 cells.

## Results

### Glucose deprivation induces VEGF expression through AhR in HepG2 cells

**Figure 1:**
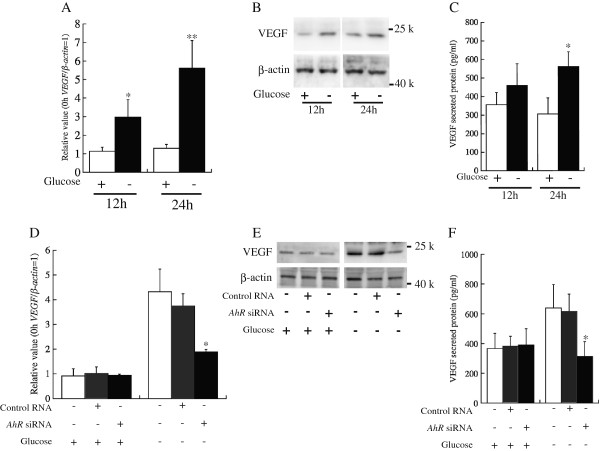
**The aryl hydrocarbon receptor (AhR) regulates vascular endothelial growth factor (VEGF) expression in glucose-deprived conditions. A** and **D**: mRNA expression of *VEGF*. **A** shows *VEGF* expression in glucose-deprived conditions, and **D** shows *VEGF* expression after *AhR* knockdown. The *VEGF* mRNA levels were normalized by the *β*-*actin* mRNA level at each point (*VEGF*/*β*-*actin*). The values of *VEGF*/*β*-*actin* were calculated relative to the expression level at 0 h (the time of medium exchange), which was set equal to 1. Bars indicate the standard deviation of independent triplicate measurements. **B** and **E**: Western blot analyses using an antibody against VEGF. + and – indicate the presence and absence, respectively, of glucose, control RNA, and *AhR* siRNA. **C** and **F**: Measurement of VEGF protein secretion to the media. “12 h” and “24 h” in **A**, **B** and **C** indicate the time following transfer to high or no glucose medium. The RNA, protein and media samples in **D**, **E** and **F** were derived from the HepG2 cells at 24 h after the medium exchange. * indicates that there is a significant difference (*: P < 0.05, **: P < 0.005).

In the HepG2 human liver carcinoma cell line, glucose deprivation, or hypoglycemia, enhances *VEGF* mRNA expression [17]. When the medium was exchanged from high glucose medium (4.5 g/L D-glucose) to no glucose medium (0 g/L), *VEGF* mRNA expression was increased in HepG2 cells at 12 and 24 h after the medium exchange (Figure 1A). Correspondingly, the expression level of protein was increased under glucose deprivation at 12 and 24 h after the medium exchange and secreted VEGF was increased under glucose deprivation at 24 h after medium exchange (Figure 1B and C).

VEGF is a potent angiogenic factor that plays a central role in angiogenesis [11], and angiogenesis is a significant step in the pre-malignancy and malignancy of cancer [21]. We expected that AhR was related to the process of cancer cell malignancy. Tryptophan-2,3-dioxygenase (TDO)-derived kynurenine promotes tumor cell survival through AhR with the progression of cell malignancy [22]. AhR is translocated to the nucleus by low glucose conditions in HepG2 cells. AhR that is translocated to the nucleus activates *CYP1A1*, *1A2*, *1B1*, and *Nrf2* expression in HepG2 cells in low glucose conditions [18]. We expected that the nucleus-translocated AhR was related to VEGF expression that was induced by glucose deprivation. When *AhR* expression was knocked down by RNAi, *VEGF* expression did not change in normal glucose conditions (D-glucose = 4.5 g/L). Under glucose deprivation (D-glucose = 0 g/L), *VEGF* expression was suppressed by RNAi against AhR (Figure 1D). The expression and secretion of VEGF protein were clearly suppressed by RNAi against *AhR* under glucose deprivation (Figure 1E and F).

### AhR and VEGF pathway analysis *in silico*

**Figure 2:**
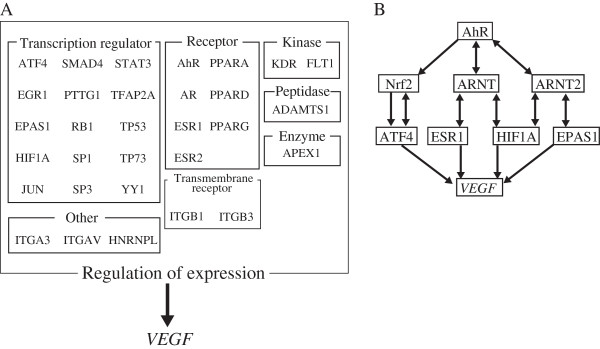
***In silico *analysis of VEGF regulation. A** shows the candidates of direct regulators of *VEGF* expression that were extracted by Ingenuity Pathway Analysis (IPA). **B** shows the pathway that was predicted by *in silico* analysis from AhR to *VEGF* expression. The direction of the lines indicates the direction of the transcriptional regulation, and double-headed arrows indicate protein-protein interactions.

Figure 1D, E and F show that AhR is required for increased expression of VEGF in HepG2 cells in glucose deprived-conditions. However, there are no AhR binding regions on the transcriptional regulatory domain of *VEGF*. Therefore, we expected that AhR did not regulate VEGF expression directly. We tried to extract candidate *VEGF* transcriptional regulators that could be induced by glucose deprivation through *in silico* analyses using Ingenuity Pathway Analysis (IPA). We extracted the molecules that were classified as “regulation of expression” and could possibly regulate *VEGF* expression directly (Figure 2A). Moreover, the candidate molecules of interest were those molecules that could be integrated into the pathway where AhR was a starting molecule and *VEGF* was at the end of the pathway. As a result, ATF4, estrogen receptor 1 (ESR1), and endothelial PAS domain protein 1 (EPAS1) were integrated into the AhR to VEGF pathway (Figure 2B). In low glucose conditions, AhR induced *Nrf2* expression [18]. Similarly, in no glucose conditions, AhR induced *Nrf2* expression (data not shown). In nuclei, phosphorylated Nrf2 protein induced the expression of ATF4 protein [23] and bound to ATF4 protein [24,25]. We expected that ATF4 has the high possibility for interacting with AhR through Nrf2 and inducing VEGF expression. Therefore, we focused on the ATF4 interaction with Nrf2.

### AhR regulates ATF 4 expression under glucose deprivation in HepG2 cells

**Figure 3:**
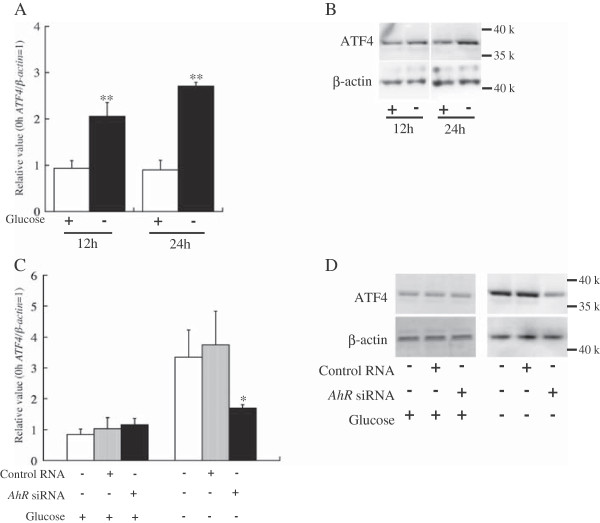
**AhR regulates activating transcription factor 4 (ATF4) expression in glucose**-**deprived conditions. A** and **C**: mRNA expression of *ATF4*. **A** shows *ATF4* expression in glucose-deprived conditions, and **C** shows *ATF4* expression after *AhR* knockdown. The *ATF4* mRNA levels were normalized by the *β*-*actin* mRNA level at each point (*ATF4*/*β*-*actin*). The *ATF4*/*β*-*actin* values were calculated relative to the expression level at 0 h (**A**: the time of medium exchange, **C**: the time of knockdown), which was set equal to 1. Bars indicate the standard deviation of independent triplicate measurements. * indicates that there is a significant difference (*: P < 0.05, **: P < 0.005). **B** and **D**: Western blot analyses using an antibody against ATF4. + and – indicate presence and absence, respectively, of glucose, control RNA, and *AhR* siRNA. “12 h” and “24 h” in **A** and **B** indicate the time following transfer to high or no glucose medium. The RNA and protein samples in **C** and **D** were derived from the HepG2 cells at 24 h after the medium exchange.

The *ATF4* transcript level was higher after 12 and 24 h of culture in glucose-deprived conditions than in high-glucose conditions (Figure 3A). The protein levels differed between glucose deprivation and high glucose after 24 h of cell culture, but they did not differ much after 12 h of culture (Figure 3B). There are no reports that AhR regulates *ATF4* expression directly. According to our current *in silico* analysis, AhR possibly regulates *ATF4* expression through Nrf2, FOS, CCAAT enhancer-binding protein alpha (CEBPA), MAF, and p53. In HepG2 cells, *AhR* knockdown suppressed *ATF4* expression in glucose-deprived conditions (Figure 3C), and the expression level of ATF4 protein was suppressed by *AhR* knockdown (Figure 3D). These results indicate that ATF4 is included in the AhR pathway and that AhR regulates ATF4 expression.

### ATF4 but not Nrf2 mediates the induction of VEGF in glucose deprivation

**Figure 4:**
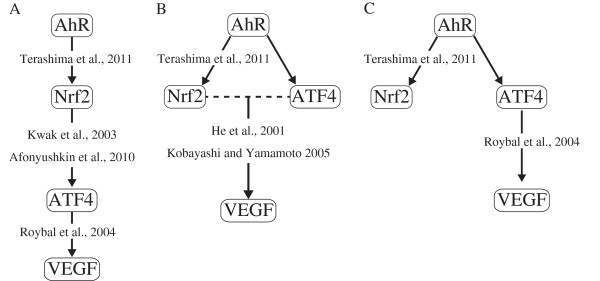
**Hypotheses of the relationships between AhR, nuclear factor erythroid-derived 2-related factor 2 (Nrf2), ATF4, and VEGF.** The hypotheses by IPA and past reports are raised (**A**, **B** and **C**, see the text for detail). Solid arrows indicate the activation of gene expression, and the dotted line indicates a protein-protein interaction.

ATF4 was shown to induce the stress-induced expression of VEGF [26]. In addition, ATF4 regulates VEGF expression in stress-induced angiogenesis, which interacts with Nrf2 [27]. In HepG2 cells, the AhR pathway regulates the stress-induced expression of *Nrf2*[18]. Therefore, we raise the following 3 hypotheses: A) Nrf2 induces VEGF expression by activating ATF4 expression; B) protein interactions between Nrf2 and ATF4 induce VEGF expression; and C) ATF4 activates VEGF expression independent of Nrf2 in stress-induced angiogenesis (Figure 4).

**Figure 5:**
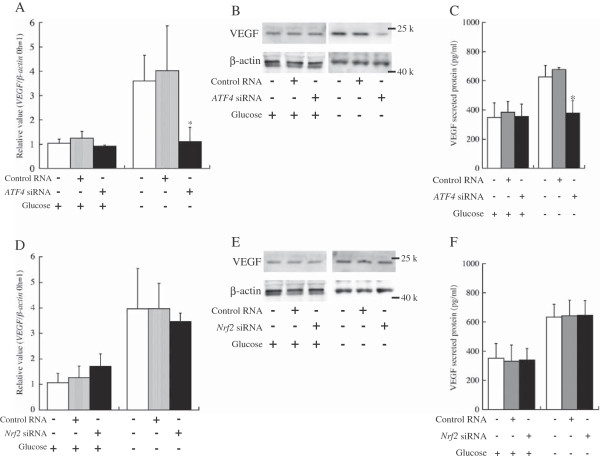
**VEGF expression under glucose deprivation is induced by ATF4 but not Nrf2. A** and **D**: mRNA expression of *VEGF*. **A** shows *VEGF* expression after *ATF4* knockdown, and **D** shows *VEGF* expression after *Nrf2* knockdown. The *VEGF* mRNA levels were normalized by the *β*-*actin* mRNA level at each point (*VEGF*/*β*-*actin*). The *VEGF*/*β*-*actin* values were calculated relative to the expression level at 0 h (the time of knockdown), which was set equal to 1. Bars indicate the standard deviation of independent triplicate measurements. **B** and **E**: Western blot analyses using an antibody against VEGF. + and – indicate the presence and absence, respectively, of glucose, control RNA, and *ATF4* or *Nrf2* siRNA. **C** and **F**: Measurement of VEGF protein secretion to the media. All RNA, protein and media amples were derived from HepG2 cells at 24 h after knockdown. * indicates that there is a significant difference (*: P < 0.05, **: P < 0.005).

**Figure 6:**
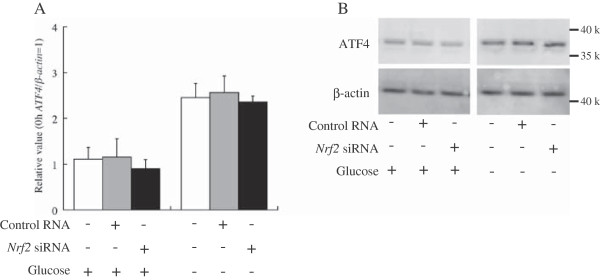
**Nrf2 is not related to ATF4 expression. A** shows *ATF4* expression after *Nrf2* knockdown. The *ATF4* mRNA levels were normalized by the *β*-*actin* mRNA level at each point (*ATF4*/*β*-*actin*). The *ATF4*/*β*-*actin* values were calculated relative to the expression level at 0 h (the time of knockdown), which was set equal to 1. Bars indicate the standard deviation of independent triplicate measurements. * indicates that there is a significant difference (*: P < 0.05, **: P < 0.005). **B** shows western blot analyses using an antibody against ATF4. + and – indicate the presence and absence, respectively, of glucose, control RNA, and *Nrf2* siRNA. All RNA or protein samples were derived from HepG2 cells at 24 h after knockdown.

Glucose deprivation-induced expression of VEGF was examined using RNAi against ATF4 or Nrf2 at the mRNA and protein levels (Figure 5). When HepG2 cells were cultured under high glucose conditions, RNAi against *ATF4* did not affect VEGF expression. However, when HepG2 cells were cultured under glucose-deprived conditions, ATF4 knockdown suppressed VEGF expression and secretion (Figure 5A,B and C). In contrast, *Nrf2* knockdown did not affect VEGF expression at the mRNA and protein levels and secretion of the VEGF protein (Figure 5D,E and F). No appreciable change in ATF4 expression at the mRNA and protein levels occurred by *Nrf2* knockdown regardless of the glucose concentration (Figure 6A and B).

## Discussion

Lines of evidence suggest that AhR has important roles in cancer pathogenesis, promotion, and malignancy [28,29]. The activation of AhR by a typical agonist, 2,3,7,8-tetrachlorodibenzo-*p*-dioxin (TCDD), has been shown to promote tumor formation in both liver and skin [30]. TCDD-mediated liver tumor promotion in multistage rat hepatocarcinogenesis has been studied in detail because of its relevance in xenobiotic-induced toxicity [31]. The expression of mutant AhR molecules by mice and rats suggested that AhR plays a key role in liver tumor promotion [32,33]. In addition, kynurenine acts as an endogenous ligand of the human AhR, and the TDO-AhR pathway is associated with the malignant progression of human brain tumors [22]. Takeuchi et al. and Rhoman et al. have shown that AhR has a role for VEGF expression and angiogenesis in mouse [19,20]. Our results indicate that AhR regulates VEGF expression through ATF4 in glucose deprived-conditions in HepG2 cells (Figures. 2, 3, and 5). VEGF is an important molecule in angiogenesis for tumor malignancy during carcinogenesis [11]. Angiogenesis is well known as one of the malignancy stages of cancer progression. Shweiki et al. raised the possibility of inducing VEGF expression by hypoxia and glucose deprivation in C6 cells, a clonal glial cell line that is derived from a rat glial tumor [34]. Several studies have demonstrated that glucose deprivation also induces VEGF expression in different types of cells [35-37]. Yun et al. reported that glucose transporter 1 (GLUT1) and 6-phosphofructo-2-kinase/fructose-2,6-bisphosphatase-3 (PFKFB3) are important molecules for VEGF expression under glucose deprived-conditions in DU145 cells, a human prostate carcinoma cell line [37]. GLUT1 and PFKFB3 are upregulated by hypoxia-inducible factor 1 (HIF1) [38,39]. Therefore, the VEGF pathway in glucose deprivation is possibly linked to a hypoxia response pathway.

Cancer cells in poorly vascularized solid tumors are constantly or intermittently exposed to glucose deprivation as well as hypoxia [40]. Glucose deprivation activates the unfolded protein response (UPR), a stress-signaling pathway in tumor cells [41]. The UPR induces the expression of the transcription factor ATF4 through PERK [42]. Glucose deficiency or deprivation induces AhR translocation into the nucleus, and the nuclear translocation of AhR activates not only CYP1A1 and CYP1A2 expression [18] but also ATF4 expression (Figure 3C). However, there are no binding elements for AhR in the ATF4 transcriptional regulatory domains, suggesting that other molecules mediate AhR-ATF4 regulation. AhR forms a dimer with ARNT, and the AhR-ARNT complex can bind to dioxin-responsive elements to activate the transcription of various genes, including *CYP1A1* and *1A2*[43,44]. Tumor cells in the microenvironment of a solid tumor are exposed to hypoxia and/or nutrient deprivation. In the microenvironments within a solid tumor, angiogenesis is triggered by the expression of various molecules including VEGF.

To understand the response of liver cancer cells to nutrient deprivation, we propose 3 hypotheses for the AhR-regulated pathway that induces VEGF in glucose-deprived HepG2 cells: A) Nrf2 induces VEGF expression by activating ATF4 expression; B) protein interactions between Nrf2 and ATF4 induce VEGF expression; and C) ATF4 activates VEGF expression independent of Nrf2 (Figure 4).

Glucose deprivation-induced expression of VEGF was examined at the mRNA and protein levels using RNAi against *ATF4* or *Nrf2* (Figure 5). When HepG2 cells were cultured under glucose deprivation, *ATF4* knockdown suppressed *VEGF* expression (Figure 5A). In contrast, *Nrf2* knockdown did not affect VEGF expression at the mRNA and protein levels and secretion of the VEGF protein (Figure 5D,E and F). No appreciable change in ATF4 expression at the mRNA and protein levels resulted from Nrf2 knockdown regardless of the glucose concentration (Figure 6). These results clearly indicated that ATF4 activated VEGF expression independent of Nrf2 in the glucose-deprived HepG2 cells. We adopt hypothesis C as illustrated in Figure 4.

## Conclusions

The AhR is well known as a ligand-activated transcription factor and transcriptional regulator for drug-metabolizing enzyme, *CYP1* family genes. AhR translocates into the nucleus following ligand binding and forms a complex with ARNT. The AhR-ARNT complex binds to the XRE domain to regulate target gene expression. In conclusion, the AhR pathway, which involves ATF4, induces VEGF expression in glucose-deprived human hepatoblastoma HepG2 cells. AhR translocates into the nucleus in response glucose deprivation in HepG2 and regulates gene expression. However, there are no binding domains of AhR on the transcriptional regulatory domain of *ATF4*. We expect that there are some mediators between the AhR translocation into the nucleus and *ATF4* expression. The pathway from AhR to VEGF through ATF4 is a novel pathway in glucose-deprived liver cancer cells, which is related to the microenvironment within cancer tissue that affects liver cancer malignancy.

## Methods

### Cell culture

HepG2 (a human hepatocellular carcinoma, ATCC No.: HB-8065) cells (1.0 × 10^5^ cells/mL) were cultured in Dulbecco’s modified Eagle’s medium (DMEM; high glucose: 4.5 g/L D-glucose; Gibco, Grand Island, NY, USA) supplemented with 10% fetal bovine serum (Hyclone, South Logan, VT, USA, Lot FRC25965) and antibiotics (Gibco) at 37°C in a CO_2_ incubator for 48 h as a pre-culture. After the pre-culture, the media were changed to high or low glucose (1.0 g/L D-glucose, Gibco) DMEM.

### RNA extraction and quantitative reverse-transcription polymerase chain reaction (qRT-PCR)

Total RNA was extracted from 3 independent HepG2 cell cultures using the RNeasy Mini Kit following the manufacturer’s instructions (Qiagen, Dusseldorf, Germany). Total RNAs (1 μg) were used for cDNA synthesis with a cDNA synthesis kit (Roche, Basel, Switzerland). Using the first-strand cDNA, real-time PCR was performed with a 7500 Real-Time PCR System (Applied Biosystems, Tokyo, Japan).

### RNA interference (RNAi) assay

The small interfering RNAs (siRNAs) against *AhR*, *Nrf2*, and *ATF4* and control siRNAs were supplied as Stealth RNAs by Invitrogen (Carlsbad, CA, USA). The siRNA sequences targeting human *AhR*, *Nrf2*, and *ATF4* mRNAs were designed. In addition, negative control siRNAs were designed by scrambling the nucleotide sequence (scramble sequence) of the siRNAs for human *AhR*, *ATF4*, and *Nrf2*. The negative control siRNAs were designed without homology to any other gene.

HepG2 cells were individually transfected with siRNAs with lipofectamine (Invitrogen) for 48 h according to the manufacturer’s instructions. All siRNA transfections were performed by treating the cells with 10 nM siRNA. The negative control group was treated with the same concentration of the negative control siRNA. All alternative control groups were treated with lipofectamine in the absence of siRNA. The HepG2 cells were cultured for 48 h in high glucose conditions as a pre-culture with siRNA or control siRNA. The suppression efficiency of gene expressions show in Additional file 1: Figure S1.

### Western blotting

HepG2 cells were lysed and separated into the cytosol and nuclear fractions using the NER-PER Nuclear and Cytoplasmic Extraction Reagent (Thermo Scientific, Rockford, IL, USA). The protein samples were subjected to sodium dodecyl sulfate 10% polyacrylamide gel electrophoresis (10 μg protein) and transferred to a nitrocellulose membrane. The blots were blocked with Chemiluminescent Blocker (Millipore, Billerica, MA, USA) at room temperature for 1 h and rinsed with tris-buffered saline containing 0.1% Tween 20 (TBS-T). The blots were incubated overnight with antibodies against VEGF (1:1000, SC-7269 Santa Cruz), ATF4 (1:1000, SC-200, Santa Cruz), or *β-actin* (1:1000, SC-130656, Santa Cruz) at 4°C, followed by washing with TBS-T. The blot was subsequently incubated for 1.5 h with a mouse-IgG (1:1000, ZYMED) secondary antibody in Chemiluminescent Blocker at room temperature followed by washes with TBS-T. After a chemiluminescent reaction using Luminate Classico Western HRP Substrate (Millipore), the bands were visualized with a LAS-3000 (Fujifilm, Tokyo, Japan).

### Quantitation of VEGF protein secretion

Media were collected from 300,000 cells in 60 mm dishes and centrifuged at 2000 r.p.m. for 10 min at 4°C. VEGF in the medium was measured by using the Quantikine human VEGF ELISA kit from R&D Systems (Minneapolis, MN, USA) according to manufacturer’s instruction.

### Analysis of the AhR-VEGF pathway by ingenuity pathway analysis

We used an Ingenuity Pathway Analysis tool to search the biological pathway from AhR to *VEGF* including the molecules involved in the pathway. We used this tool to identify candidate molecules that were glucose deprivation-induced *VEGF* transcriptional regulators in conjunction with biological pathways that are activated by AhR.

## Abbreviations

AhR: Aryl hydrocarbon receptor; VEGF: Vascular endothelial growth factor; ATF4: Activating transcription factor 4; ARNT: AhR nuclear translocator; Nrf2: Nuclear factor erythroid-derived 2-related factor 2; CYP: Cytochrome P450.

## Competing interests

The authors declare that they have no competing interests.

## Authors’ contributions

JT, HW, KK and SO conceived and designed the experiments and analyzed the data. JT, CT, and SO wrote the manuscript: JT and CT performed the experiments. All authors read and approved the final manuscript.

## Supplementary Material

Additional file 1: Figure S1 Suppresseion eficiency of gene expressions by RNAi. The graphs show *AhR*, *ATF4* and *Nrf2* expressions under addition of siRNA for *AhR*, *ATF4* and *Nrf2* respectively. Each mRNA levels were normalized by the *β*-*actin* mRNA level at each point (*AhR*, *ATF4* or *Nrf2*/*β*-*actin*). The values of *AhR*, *ATF4* or *Nrf2*/*β*-*actin* were calculated relative to the expression level at 0 h (the time of medium exchange), which was set equal to 1. Bars indicate the standard deviation of independent triplicate measurements. * indicates that there is a significant difference (*: P < 0.05, **: P < 0.005). Click here for file

## References

[B1] Denson MS Nagy SR Activation of the aryl hydrocarbon receptor by structurally diverse exogenous and endogenous chemicals Annu Rev Pharmacol Toxicol 2003 43 309 334 10.1146/annurev.pharmtox.43.100901.135828 12540743

[B2] Fujii-Kurihara Y Miura J Molecular mechanisms of AhR functions in the regulation of cytochrome P450 genes Biochem Biophys Res Commun 2005 338 311 317 10.1016/j.bbrc.2005.08.162 16153594

[B3] Kimura S Gonzalez FJ Nebert DW Tissue-specific expression of the mouse dioxin-inducible P(1)450 and P(3)450 genes: differential transcriptional activation and mRNA stability in liver and extrahepatic tissues Mol Cell Biol 1986 6 1471 1477 3785172 10.1128/mcb.6.5.1471-1477.1986 PMC367672

[B4] Gonzalez FJ Nebert DW Autoregulation plus upstream positive and negative control regions associated with transcriptional activation of the mouse P1(450) gene Nucleic Acids Res 1985 13 7269 7288 10.1093/nar/13.20.7269 2997746 PMC322043

[B5] Fletcher N Wahlström D Lundberg R Nilsson CB Nilsson KC Stockling K Hellmold H Håkansson H 2,3,7,8-Tetrachlorodibenzo-p-dioxin (TCDD) alters the mRNA expression of critical genes associated with cholesterol metabolism, bile acid biosynthesis, and bile transport in rat liver: a microarray study Toxicol Appl Pharmacol 2005 207 1 24 16054898 10.1016/j.taap.2004.12.003

[B6] Boverhof DR Burgoon LD Tashiro C Chittim B Harkema JR Jump DB Zacharewski TR Temporal and dose-dependent hepatic gene expression patterns in mice provide new insights into TCDD-Mediated hepatotoxicity Toxicol Sci 2005 85 1048 1063 10.1093/toxsci/kfi162 15800033

[B7] Kennedy LH Sutter CH Leon Carrion S Tran QT Bodreddigari S Kensicki E Mohney RP Sutter TR 2,3,7,8-Tetrachlorodibenzo-p-dioxin-mediated production of reactive oxygen species is an essential step in the mechanism of action to accelerate human keratinocyte differentiation Toxicol Sci 2013 132 235 249 10.1093/toxsci/kfs325 23152189 PMC3576006

[B8] Ma Q Kinneer K Bi Y Chan JY Kan YW Induction of murine NAD(P)H:quinone oxidoreductase by 2,3,7,8-tetrachlorodibenzo-p-dioxin requires the CNC (cap 'n' collar) basic leucine zipper transcription factor Nrf2 (nuclear factor erythroid 2-related factor 2): cross-interaction between AhR (aryl hydrocarbon receptor) and Nrf2 signal transduction Biochem J 2004 377 205 213 10.1042/BJ20031123 14510636 PMC1223846

[B9] Miao W Hu L Scrivens PJ Batist G Transcriptional regulation of NF-E2 p45-related factor (NRF2) expression by the aryl hydrocarbon receptor-xenobiotic response element signaling pathway: direct cross-talk between phase I and II drug-metabolizing enzymes J Biol Chem 2005 280 20340 20348 10.1074/jbc.M412081200 15790560

[B10] Ichihara S Yamada Y Gonzalez FJ Nakajima T Murohara T Ichihara G Inhibition of ischemia-induced angiogenesis by benzo[a]pyrene in a manner dependent on the aryl hydrocarbon receptor Biochem Biophys Res Commun 2009 381 44 49 10.1016/j.bbrc.2009.01.187 19351592 PMC2790146

[B11] Tischer E Gospodarowicz D Mitchell R Silva M Schilling J Lau K Crisp T Fiddes JC Abraham JA Vascular endothelial growth factor: a new member of the platelet-derived growth factor gene family Biochem Biophys Res Commun 1989 165 1198 1206 10.1016/0006-291X(89)92729-0 2610687

[B12] Gunga HC Kirsch K Röcker L Behn C Koralewski E Davila EH Estrada MI Johannes B Wittels P Jelkmann W Vascular endothelial growth factor in exercising humans under different environmental conditions Eur J Appl Physiol Occup Physiol 1999 79 484 490 10.1007/s004210050541 10344456

[B13] Shweiki D Itin A Soffer D Keshet E Vascular endothelial growth factor induced by hypoxia may mediate hypoxia-initiated angiogenesis Nature 1992 359 843 845 10.1038/359843a0 1279431

[B14] Galoforo SS Berns CM Erdos G Corry PM Lee YJ Hypoglycemia-induced AP-1 transcription factor and basic fibroblast growth factor gene expression in multidrug resistant human breast carcinoma MCF-7/ADR cells Mol Cell Biochem 1996 155 163 171 8700161 10.1007/BF00229313

[B15] Lee AH Happerfield LC Bobrow LG Millis RR Angiogenesis and inflammation in invasive carcinoma of the breast J Clin Pathol 1997 50 669 673 10.1136/jcp.50.8.669 9301551 PMC500114

[B16] Spitz DR Sim JE Ridnour LA Galoforo SS Lee YJ Glucose deprivation-induced oxidative stress in human tumor cells. A fundamental defect in metabolism? Ann N Y Acad Sci 2000 899 349 362 10863552 10.1111/j.1749-6632.2000.tb06199.x

[B17] Park SH Kim KW Lee YS Baek JH Kim MS Lee YM Lee MS Kim YJ Hypoglycemia-induced VEGF expression is mediated by intracellular Ca2+ and protein kinase C signaling pathway in HepG2 human hepatoblastoma cells Int J Mol Med 2001 7 91 96 11115615

[B18] Terashima J Habano W Gamou T Ozawa S Induction of CYP1 family members under low-glucose conditions requires AhR expression and occurs through the nuclear translocation of AhR Drug Metab Pharmacokinet 2011 26 577 583 10.2133/dmpk.DMPK-11-RG-054 21878739

[B19] Takeuchi A Takeuchi M Oikawa K Sonoda KH Usui Y Okunuki Y Takeda A Oshima Y Yoshida K Usui M Goto H Kuroda M Effect of dioxin on vascular endothelial grouth factor (VEGF) production in the retina associated with choroidal neovascularization Invest 2009 50 3410 3415 10.1167/iovs.08-2299 19182260

[B20] Rhoman AC Carvajal-Gonzales JM Rico-Leo EM Fernandez-Salguero DM Dioxin receptor deficiency impairs angiogenesis by a mechanism involving VEGF-A depletion in the endothelium and transforming growth factor-β overexpression in the stroma J Biol Chem 2009 284 25135 25148 10.1074/jbc.M109.013292 19617630 PMC2757217

[B21] Bamberger ES Perrett CW Angiogenesis in benign, pre-malignant and malignant vulvar lesions Anticancer Res 2002 22 3853 3865 12553005

[B22] Opitz CA Litzenburger UM Sahm F Ott M Tritschler I Trump S Schumacher T Jestaedt L Schrenk D Weller M Jugold M Guillemin GJ Miller CL Lutz C Radlwimmer B Lehmann I Von Deimling A Wick W Platten M An endogenous tumour-promoting ligand of the human aryl hydrocarbon receptor Nature 2011 478 197 203 10.1038/nature10491 21976023

[B23] Kwak MK Wakabayashi N Itoh K Motohashi H Yamamoto M Kensler TW Modulation of gene expression by cancer chemopreventive dithiolethiones through the Keap1-Nrf2 pathway. Identification of novel gene clusters for cell survival J Biol Chem 2003 278 8135 8145 10.1074/jbc.M211898200 12506115

[B24] He CH Gong P Hu B Stewart D Choi ME Choi AM Alam J Identification of activating transcription factor 4 (ATF4) as an Nrf2-interacting protein. Implication for heme oxygenase-1 gene regulation J Biol Chem 2001 276 20858 20865 10.1074/jbc.M101198200 11274184

[B25] Kobayashi M Yamamoto M Molecular mechanisms activating the Nrf2-Keap1 pathway of antioxidant gene regulation Antioxid Redox Signal 2005 7 385 394 10.1089/ars.2005.7.385 15706085

[B26] Roybal CN Yang S Sun CW Hurtado D Vander Jagt DL Townes TM Abcouwer SF Homocysteine increases the expression of vascular endothelial growth factor by a mechanism involving endoplasmic reticulum stress and transcription factor ATF4 J Biol Chem 2004 279 14844 14852 10.1074/jbc.M312948200 14747470

[B27] Afonyushkin T Oskolkova OV Philippova M Resink TJ Erne P Binder BR Bochkov VN Oxidized phospholipids regulate expression of ATF4 and VEGF in endothelial cells via NRF2-dependent mechanism: novel point of convergence between electrophilic and unfolded protein stress pathways Arterioscler Thromb Vasc Biol 2010 30 1007 1013 10.1161/ATVBAHA.110.204354 20185790

[B28] Adams S Braidy N Bessede A Brew BJ Grant R Teo C Guillemin GJ The kynurenine pathway in brain tumor pathogenesis Cancer Res 2012 72 5649 5657 10.1158/0008-5472.CAN-12-0549 23144293

[B29] Barouki R Aggerbeck M Aggerbeck L Coumoul X The aryl hydrocarbon receptor system Drug Metabol Drug Interact 2012 27 3 8 22718620 10.1515/dmdi-2011-0035

[B30] Ray S Swanson HI Activation of the aryl hydrocarbon receptor by TCDD inhibits senescence: a tumor promoting event? Biochem Pharmacol 2009 77 681 688 10.1016/j.bcp.2008.11.022 19100242 PMC2662439

[B31] Huff J Lucier G Tritscher A Carcinogenicity of TCDD: experimental, mechanistic, and epidemiologic evidence Annu Rev Pharmacol Toxicol 1994 34 343 372 10.1146/annurev.pa.34.040194.002015 8042855

[B32] Viluksela M Bager Y Tuomisto JT Scheu G Unkila M Pohjanvirta R Flodström S Kosma VM Mäki-Paakkanen J Vartiainen T Klimm C Schramm KW Wärngård L Tuomisto J Liver tumor-promoting activity of 2,3,7,8-tetrachlorodibenzo-p-dioxin (TCDD) in TCDD-sensitive and TCDD-resistant rat strains Cancer Res 2000 60 6911 6920 11156390

[B33] Moennikes O Loeppen S Buchmann A Andersson P Ittrich C Poellinger L Schwarz M A constitutively active dioxin/aryl hydrocarbon receptor promotes hepatocarcinogenesis in mice Cancer Res 2004 64 4707 4710 10.1158/0008-5472.CAN-03-0875 15256435

[B34] Shweiki D Neeman M Itin A Keshet E Induction of vascular endothelial growth factor expression by hypoxia and by glucose deficiency in multicell spheroids: implications for tumor angiogenesis Proc Natl Acad Sci U S A 1995 92 768 772 10.1073/pnas.92.3.768 7531342 PMC42701

[B35] Stein I Neeman M Shweiki D Itin A Keshet E Stabilization of vascular endothelial growth factor mRNA by hypoxia and hypoglycemia and coregulation with other ischemia-induced genes Mol Cell Biol 1995 15 5363 5368 7565686 10.1128/mcb.15.10.5363 PMC230785

[B36] Zhang W Ran S Sambade M Huang X Thorpe PE A monoclonal antibody that blocks VEGF binding to VEGFR2 (KDR/Flk-1) inhibits vascular expression of Flk-1 and tumor growth in an orthotopic human breast cancer model Angiogenesis 2002 5 35 44 10.1023/A:1021540120521 12549858

[B37] Yun H Lee M Kim SS Ha J Glucose deprivation increases mRNA stability of vascular endothelial growth factor through activation of AMP-activated protein kinase in DU145 prostate carcinoma J Biol Chem 2005 280 9963 9972 10.1074/jbc.M412994200 15640157

[B38] Semenza GL Regulation of mammalian O2 homeostasis by hypoxia-inducible factor 1 Annu Rev Cell Dev Biol 1999 15 551 578 10.1146/annurev.cellbio.15.1.551 10611972

[B39] Wenger RH Cellular adaptation to hypoxia: O2-sensing protein hydroxylases, hypoxia-inducible transcription factors, and O2-regulated gene expression FASEB J 2002 16 1151 1162 10.1096/fj.01-0944rev 12153983

[B40] Acker T Plate KH A role for hypoxia and hypoxia-inducible transcription factors in tumor physiology J Mol Med (Berl) 2002 80 562 575 10.1007/s00109-002-0355-1 12226738

[B41] Kaufman RJ Orchestrating the unfolded protein response in health and disease J Clin Invest 2002 110 1389 1398 12438434 10.1172/JCI16886 PMC151822

[B42] Harding HP Novoa I Zhang Y Zeng H Wek R Schapira M Ron D Regulated translation initiation controls stress-induced gene expression in mammalian cells Mol Cell 2000 6 1099 1108 10.1016/S1097-2765(00)00108-8 11106749

[B43] Kewley RJ Whitelaw ML Chapman-Smith A The mammalian basic helix-loop-helix/PAS family of transcriptional regulators Int J Biochem Cell Biol 2004 36 189 204 10.1016/S1357-2725(03)00211-5 14643885

[B44] Nebert DW Karp CL Endogenous functions of the aryl hydrocarbon receptor (AHR): intersection of cytochrome P450 1 (CYP1)-metabolized eicosanoids and AHR biology J Biol Chem 2008 283 36061 36065 10.1074/jbc.R800053200 18713746 PMC2606007

