# Supplementary material for: An aryl hydrocarbon receptor induces VEGF expression through ATF4 under glucose deprivation in HepG2
Source: BMC Mol Biol. 2013 Dec 12;14:27. doi: 10.1186/1471-2199-14-27 (PMC3866938; doi:10.1186/1471-2199-14-27)
Supplement: Additional file 1: Figure S1 — Suppresseion eficiency of gene expressions by RNAi. The graphs show AhR, ATF4 and Nrf2 expressions under addition of siRNA for AhR, ATF4 and Nrf2 respectively. Each mRNA levels were normalized by the β-actin mRNA level at each point (AhR, ATF4 or Nrf2/β-actin). The values of AhR, ATF4 or Nrf2/β-actin were calculated relative to the expression level at 0 h (the time of medium exchange), which was set equal to 1. Bars indicate the standard deviation of independent triplicate measurements. * indicates that there is a significant difference (*: P < 0.05, **: P < 0.005). [file 1471-2199-14-27-S1.pdf]

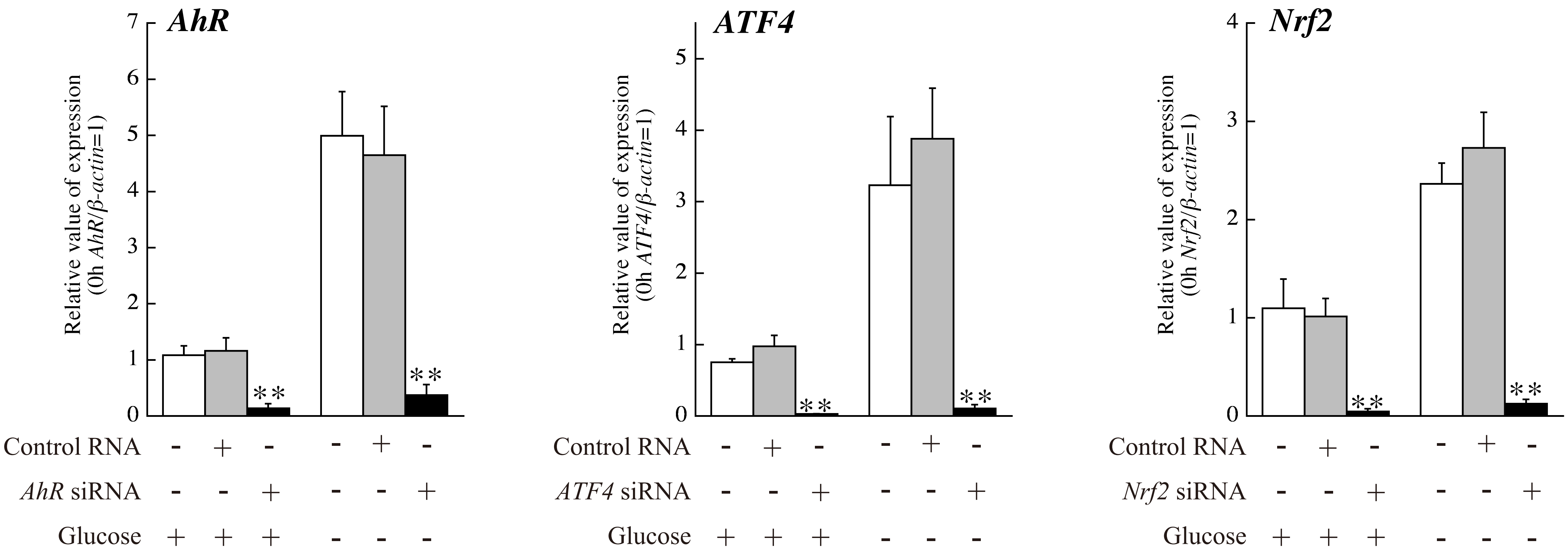


**Supplemental data 1: Suppresseion eficiency of gene expressions by RNAi**

The graphs show *AhR, ATF4* and *Nrf2* expressions under addition of siRNA for *AhR, ATF4* and *Nrf2* respectively. Each mRNA levels were normalized by the *β-actin* mRNA level at each point (*AhR, ATF4* or *Nrf2*/*β-actin*). The values of *AhR, ATF4* or *Nrf2*/*β-actin* were calculated relative to the expression level at 0 h (the time of medium exchange), which was set equal to 1. Bars indicate the standard deviation of independent triplicate measurements.

* indicates that there is a significant difference (*: $P < 0.05$, **: $P < 0.005$).
